# Career‐stage patterns in burnout and intention to leave the current hospital among obstetric physicians

**DOI:** 10.1111/aogs.70274

**Published:** 2026-06-16

**Authors:** Arno Stöcker, Malte Bäckmann, Matthias Nübling, Mi‐Ran Okumu, Brigitte Strizek, Anna Volkert, Matthias Weigl, Nadine Scholten

**Affiliations:** ^1^ Center for Health Services Research and Communication, Department for Psychosomatic Medicine and Psychotherapy, Faculty of Medicine University Hospital Bonn Bonn Germany; ^2^ Freiburg Research Center for Occupational Sciences Freiburg Germany; ^3^ Department of Obstetrics and Prenatal Medicine University Hospital Bonn Bonn Germany; ^4^ Institute for Patient Safety University Hospital Bonn Bonn Germany; ^5^ Chair of Health Services Research, Institute of Medical Sociology, Health Services Research and Rehabilitation Science, Faculty of Medicine and University Hospital Cologne University of Cologne Cologne Germany

**Keywords:** career mobility, clinical workforce, Copenhagen Burnout Inventory, health workforce retention, occupational well‐being, physician turnover

## Abstract

**Introduction:**

To examine associations between personal burnout symptoms and the intention to leave the current hospital across different career stages among physicians in obstetric care in Germany. In addition, we compared burnout levels among surveyed obstetricians relative to the general physician and the German working population.

**Material and Methods:**

In this nationwide, cross‐sectional study, physicians from all 595 obstetric departments in Germany were invited to complete an anonymous, standardized survey. We assessed burnout, intentions to leave the workplace within the next 5 years, career stage, age, gender, and employment status. Comparative data on burnout were obtained from a national database on mental stress in the workplace. Data were analyzed visually using Sankey plots and Firth‐corrected multivariable logistic regression.

**Results:**

A total of 872 obstetricians responded. Overall, 380 physicians (50.9%) reported intention to leave the current hospital within the next 5 years. Moderate personal burnout was reported by 332 physicians (40.2%), while 168 (20.4%) reported high or severe burnout. Average burnout levels among surveyed obstetricians (mean: 50.6) were comparable to those of other physicians (mean: 51.4) and the general working population (mean: 49.9) in Germany. Intentions to leave were most frequent among residents (176, 74.2%), followed by specialists (46, 57.5%). Regression analyses showed that higher burnout scores were significantly associated with intention to leave the current hospital (OR = 1.03, 95%‐CI: 1.02, 1.04, *p* < 0.001), particularly among physicians in earlier career stages. With each one‐point increase in burnout scores, odds of intending to leave increased by 3.3% for residents (95%‐CI: 1.02, 1.05, *p* < 0.001), 3.2% for specialists (95%‐CI: 1.01, 1.06, *p* = 0.005), and 3.2% for consultants without executive responsibilities (95%‐CI: 1.02, 1.05, *p* < 0.001).

**Conclusions:**

Personal burnout symptoms are significantly associated with the intention to leave the current hospital, particularly among early‐career obstetricians. While career‐stage–related mobility can partly explain turnover, burnout appears to be an independent determinant. Our findings highlight the importance of addressing burnout early in medical careers to support physician retention in obstetric care.

## Graphical abstract

Obstetric physicians across all career stages reported burnout symptoms and intention to leave their current hospital, with varying patterns by professional position. These findings highlight the importance of career‐stage–specific retention and wellbeing strategies.

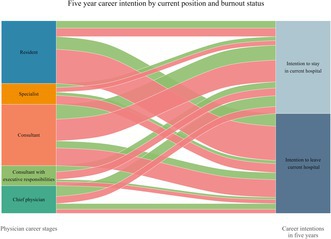

**Abbreviations**
CBI: Copenhagen Burnout Inventory
ITL: intention to leave
ITLCH: intention to leave current hospital
OBGYN: obstetrics and gynecology

**Key message**

Burnout is significantly associated with an increased intention to leave the current hospital, particularly among early‐career obstetricians. These findings emphasize the need for targeted retention initiatives and the implementation of career‐stage–specific strategies to mitigate burnout and enhance workforce stability.

## 1 INTRODUCTION

Physicians' intentions to leave (ITL) their employing healthcare organization threaten workforce retention, continuity, and quality of care.^1^, ^2^ ITL is particularly consequential amid staff shortages, an aging workforce, and rising demand for specialized services,^1^ and is a strong predictor of actual turnover.^3^

Burnout, defined as a state of physical, emotional, and mental exhaustion from prolonged workplace stress,^4^ is a key driver of ITL,^5^ encompassing both the desire to leave the medical profession altogether and the intention to leave one's current place of employment. Among physicians, burnout is associated with reduced care quality and medical errors,^6^, ^7^, ^8^ and career dissatisfaction and increased likelihood of leaving the job or profession,^5^, ^8^, ^9^ causing organizational and economic strain.^10^

Burnout^11^ and ITL^3^, ^12^ are especially prevalent in obstetrics and gynecology (OBGYN), reflecting high workloads, regulatory pressures, limited autonomy, and work–life imbalance.^7^ Furthermore, inpatient obstetric care is characterized by frequent exposure to traumatic events, which add substantially to work‐related stressors.^13^ Especially, burnout is higher among early‐career and younger physicians.^8^, ^14^, ^15^, ^16^ While burnout is known to predict ITL,^8^, ^12^ how this relationship varies across career stages remains unclear.^15^, ^17^ Understanding these interactions between burnout and intention to leave the current hospital (ITLCH) is crucial, as retention efforts may need to be tailored to career‐specific stressors and expectations.^6^ Early‐career physicians face workload pressures and limited control,^15^, ^16^ whereas mid‐ and late‐career physicians encounter administrative demands, leadership responsibilities, and retirement considerations.^16^

This study examines burnout and ITLCH among OBGYN physicians in German hospitals, focusing on differences across career stages. Understanding these dynamics is vital for developing targeted retention strategies that address career‐specific stressors.

## 2 MATERIAL AND METHODS

### 2.1 Design

A nationwide, cross‐sectional survey was conducted in Germany in 2023 as part of a project on needs, participation, and safety in obstetric care (MAM‐Care), funded by the Federal Ministry of Research, Technology and Space (funding no. 01GY2110).

Recruiting was based on a nationwide list of all OBGYN departments. In Germany, 98% of all births take place in inpatient obstetric departments, which are mostly integrated into general hospitals and required to publish annual quality reports. Based on these legally mandated reports, contact data for all obstetric departments were compiled and reviewed, resulting in a validated, Germany‐wide list of 595 OBGYN departments.

Every chief of an obstetrics department received personalized mailings containing a personalized cover letter, study information, paper‐based questionnaires for themselves and their team, and prepaid return envelopes. Leaflets included a QR code for optional online participation. Furthermore, the survey was distributed via the newsletter of the Working Group for Obstetrics and Prenatal Medicine of the German Society for Gynecology and Obstetrics and its Young Forum. Participation in the anonymous survey was considered to imply informed consent upon return of the completed questionnaire or submission of the online survey. Responses could not be linked to individual hospitals, and no personally identifiable information was collected. Because the survey was disseminated using a distribution model that prevented identification of the total number of eligible individuals, calculation of a survey response rate was not possible.

### 2.2 Measures

Physicians working in obstetrics were involved in the development of the survey as well as in carrying out a cognitive pre‐test of the questionnaire. After the return, questionnaires were automatically scanned for data entry and subsequently merged with data retrieved through the web‐based survey. Final data for the analysis is available here: https://osf.io/vxc7h/overview?view_only=aa777033768943f889f78916393befb1. Table 1 reports detailed measures on survey tools.

**TABLE 1:** Descriptive statistics on study population (*N* = 872) and description of survey questions.

Measures	*N* = 872, *N* (%)	CBI/ITLCH mean	Description/survey questions
**Career intentions in 5 years**			*“Please indicate where you think you will be professionally in 5 years? In 5 years I will be…”* *Final aggregation:* *Intention to stay (in the same hospital: same or different position); n = 367* *Intention to leave (in a different hospital: same position, in a different hospital: different position, in the outpatient sector: self‐employed, in the outpatient sector: employed, no longer working in the medical sector); n = 380*
In the same hospital: same position	275 (36.8%)	43.70/–	
In the same hospital: different position	92 (12.3%)	48.81/–	
In a different hospital: same position	40 (5.4%)	52.78/–	
In a different hospital: different position	91 (12.2%)	55.86/–	
In the outpatient sector: self‐employed	92 (12.3%)	58.51/–	
In the outpatient sector: employed	91 (12.2%)	59.55/–	
No longer working in the medical sector	66 (8.8%)	47.95/–	
Missing values	125	51.53/–	
**Personal burnout score**	Mean (SD)		*The German version in the COPSOQ III version used here comprises a 3‐item scale (Lincke et al*.,^18^ *) with the following items:* *“How often are you physically exhausted?”* *“How often are you emotionally exhausted?”* *“How often do you feel worn out?”* *Measured using a 5‐point Likert scale with following scoring system: always (100), often (75), sometimes (50), seldom (25), never/almost never (0)*
*Average personal burnout score* Std. deviation	50.60 (21.09)		
*Personal burnout score classification* *			
Low (<50)	325 (39.4%)	28.13/0.41	
Moderate (50–<75)	332 (40.2%)	57.88/0.52	
High (75–<100)	154 (18.7%)	77.60/0.66	
Severe (100)	14 (1.7%)	100/0.83	
Missing values	47	–/0.44	
**Physician career stages**			*“In what function do you work as an employed physician in the hospital?”*
Residents	263 (33.0%)	54.74/0.74	
Specialists	90 (11.3%)	54.31/0.57	
Consultants	257 (32.2%)	51.41/0.40	
Consultants with executive responsibilities	81 (10.2%)	48.85/0.31	
Chief physicians	107 (13.4%)	40.09/0.36	
Missing values	74	39.58/0.29	
**Age**			*“What is your age?” [surveyed in age groups]*
30 years and younger	126 (15.4%)	55.67/0.75	
31–40 years	270 (32.9%)	55.15/0.62	
41–50 years	179 (21.8%)	50.00/0.39	
51–60 years	177 (21.6%)	46.50/0.22	
61–70 years	67 (8.2%)	37.95/0.70	
71 years and older	1 (0.1%)	25.00/1.00	
Missing values	52	38.54/0	
**Gender**			*“What is your gender?”*
Male	220 (26.8%)	40.83/0.46	
Female	600 (73.2%)	54.40/0.53	
Missing values	52	41.15/0.50	
**Employment status**			*“Are you employed full‐time (35 h or more per week) at this hospital?”*
Part‐time employment	275 (33.6%)	50.65/0.54	
Full‐time employment	543 (66.4%)	50.64/0.50	
Missing values	54	46.76/0	

* This classification has not been officially validated by the Copenhagen Psychosocial Questionnaire development team, but was deployed by Creedy et al.^20^

#### 2.2.1 Outcome variable

Participants were requested to anticipate and report on their intended, prospective professional trajectory, i.e., where they envisioned themselves in 5 years (for wording, see Table 1). Seven predefined response options were provided: (1) remain in their current hospital and position; (2) remain in the hospital setting but in a different position; (3) move to a different hospital while staying in the same position; (4) move to a different hospital and changing positions; (5) work in the sector as a self‐employed physician; (6) work in the outpatient sector as an employed physician; (7) or leave the medical profession altogether. For statistical modeling, responses were dichotomized into two categories: intention to stay in the current hospital (responses 1 to 2) and intention to leave the current hospital (responses 3 to 7).

#### 2.2.2 Predictor variables for ITLCH

Burnout was assessed with the Copenhagen Burnout Inventory Scale (CBI).^4^ It measures personal burnout as a state of prolonged physical and psychological exhaustion. A 3‐item version 18 of the German translation by Nübling et al.^19^ was used. The scale ranges from 0 to 100, with scores of 50 or above considered to indicate symptoms of burnout.^14^, ^20^ A standardized Cronbach alpha of 0.88 indicated very good internal consistency.

For physician career stages, five answer categories were given: resident, specialist, consultant, consultant with executive responsibilities, and chief physician.

We further collected personal sociodemographic information on the following variables: age (in 10‐year intervals), gender, and employment status (full‐time vs. part‐time).

### 2.3 Statistical analysis

First, descriptive and inferential statistics were applied. To illustrate how the relationship between burnout scores and ITL the current hospital manifests along different physician career stages, we opted for a visual approach using Sankey diagrams. Finally, multivariate Firth‐corrected logistic regression models were constructed to account for the high number of categorical variables and to avoid bias from empty cells.^21^ Subgroup analyses by physician career stage complement and complete the overall analysis. All analyses were conducted using only fully completed responses for the respective variables. Given the large number of categorical variables, further statistical modeling (e.g., interaction terms or stratified analyses) was not feasible without compromising interpretability and statistical power. Data preparation (tidyverse package [2.0.0]) and analysis (logistf package [1.26.1], psych package [2.4.6.26], car package [3.1–3], gt package [0.11.1] gtsummary package [2.0.4], ggalluvial package [0.12.5]) were performed in R (version 4.4.2) with RStudio (version 2024.09.01 + 394). The syntax for the analysis is available here: https://osf.io/vxc7h/overview?view_only=aa777033768943f889f78916393befb1.

## 3 RESULTS

### 3.1 Sample characteristics

A total of 872 physicians working in obstetrics responded (700 via paper, 172 online). Table 1 reports all descriptive statistics. The sample included physicians across all career stages: 33.0% residents, 11.3% specialists, 32.2% consultants, 10.2% consultants with executive responsibility, and 13.4% chief physicians. The majority of respondents were 50 years and younger (70.1%) and female (73.2%). 33.6% reported working part‐time.

The average personal burnout score was 50.6 (sd: 21.1; scale range 0–100). 60.6% of physicians reported personal burnout scores of 50 or above, indicating symptoms of personal burnout. Concerning anticipated career trajectories, 50.9% of physicians stated intentions to leave their current hospital organization within the next 5 years.

A one‐way ANOVA revealed a significant effect of physician career stages on burnout levels (F[4, 705] = 10.02, *p* < 0.001). Mean scores decreased substantially from residents (M = 54.8) and specialists (54.7) to consultants (51.5), consultants with executive responsibility (47.9), and chief physicians (39.7). Post‐hoc pairwise comparisons with Bonferroni‐adjustment indicated that chief physicians scored significantly lower than residents (*p* < 0.001), specialists (*p* < 0.001), and consultants (*p* < 0.001), while other group differences were not statistically significant.

### 3.2 Graphical analysis

**FIGURE 1:**
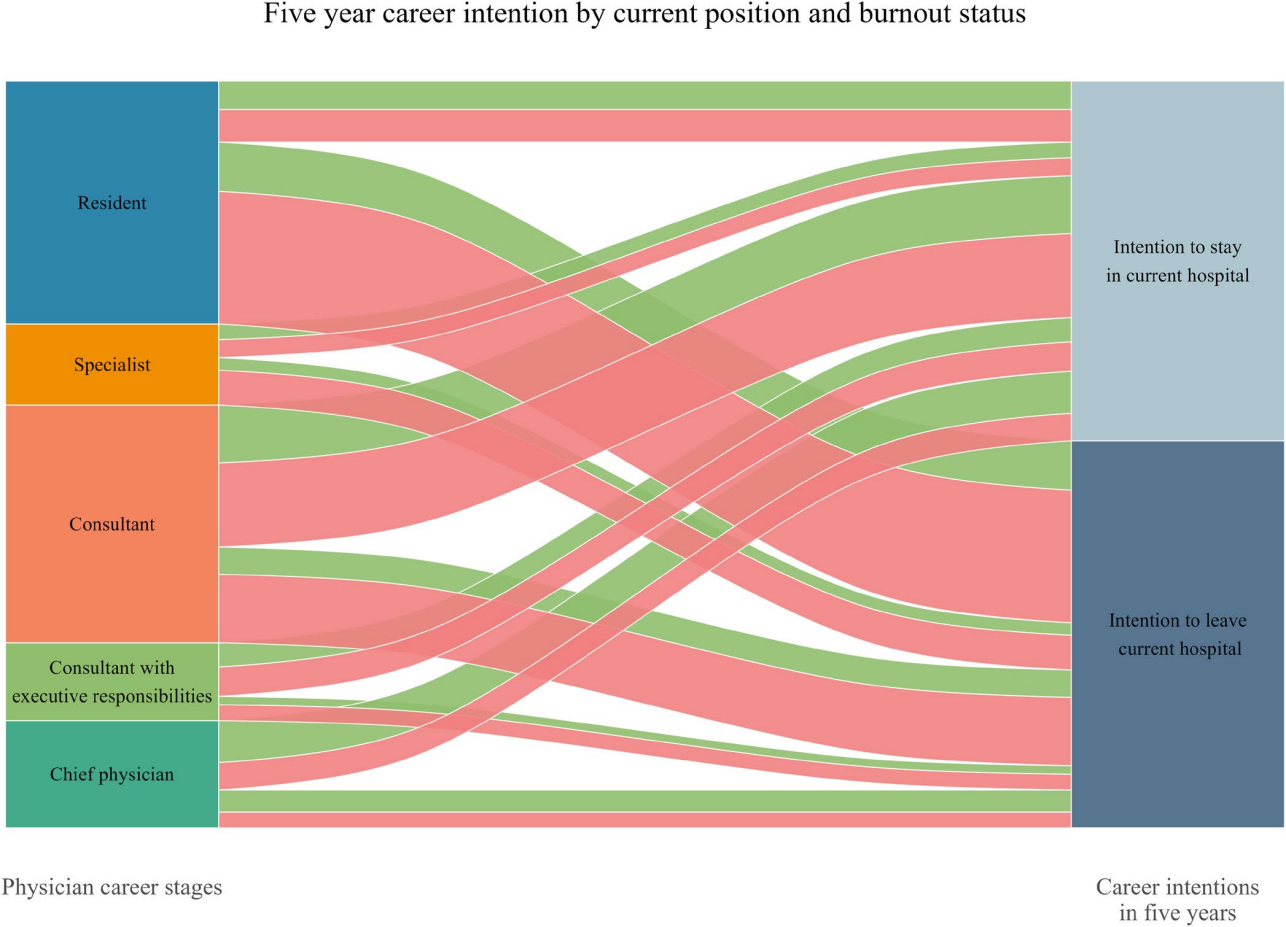
Sankey diagram illustrating the projected five‐year career intentions of physicians in OBGYN by current career stages and burnout status (*n* = 707). The left side of the diagram depicts current career stages, while the right side represents five‐year career intentions, grouped as “Intention to stay in hospital” or “Intention to leave hospital.” The width of each stream corresponds to the number of individuals following a specific path. Stream colors indicate burnout status: Red for physicians with burnout scores ≥50 and green for those with burnout scores <50.

Career intentions varied considerably across different physician career stages (Figure 1). Notably, residents were more likely to express an ITL their current organization. Specialists showed a more even distribution along possible professional career options. In contrast, consultants with and without executive responsibilities and chief physicians most frequently reported a desire to remain in their current hospital roles. Burnout (red color) was present across all career stages, but appeared visually more prevalent among those intending to leave their current hospital within the next 5 years. Particularly among residents, specialists, and consultants, the proportion with burnout scores above 50 was noticeably higher in those reporting an ITLCH compared to those without such an intention.

### 3.3 Multivariable logistic regression models

In a multivariable logistic regression model, we found a significant relationship between physicians' burnout and ITLCH (cf., Table 2). Specifically, an increase in burnout was associated with higher odds of intending to leave the current organization (OR = 1.03, *p* < 0.001). Higher‐level professional positions (i.e., chief physician) tended to be associated with lower odds of ITLCH. A similar, though less statistically robust, pattern was observed for age: with decreasing odds of ITLCH with higher age. Only physicians aged 51–60 had significantly lower odds of ITLCH compared to those aged ≤30 years (OR = 0.32, *p* = 0.006), whereas this trend reversed in the oldest age group: physicians aged 61–70 years had significantly higher odds of ITLCH compared to those aged ≤30 years (OR = 4.06, *p* = 0.005). Female sex (OR = 0.50, *p* = 0.003) and full‐time employment (OR = 0.57, *p* = 0.006) were significantly associated with lower ITLCH.

**TABLE 2:** Multivariable logistic regression model for association between burnout and ITLCH.

Predictor	OR [95% CI]
Intercept	2.23 [1.00; 5.07]
Personal burnout score (CBI 3 item)	1.03*** [1.02; 1.04]
Specialist (vs. resident)	0.52* [0.27; 0.99]
Consultant (vs. resident)	0.26*** [0.15; 0.45]
Consultant w. exec. resp. (vs. resident)	0.16*** [0.07; 0.35]
Chief physician (vs. resident)	0.19*** [0.08; 0.43]
Age 31–40 (vs. ≤30)	0.74 [0.39; 1.36]
Age 41–50 (vs. ≤30)	0.59 [0.27; 1.29]
Age 51–60 (vs. ≤30)	0.32** [0.14; 0.73]
Age 61–70 (vs. ≤30)	4.06** [1.51; 11.16]
Female (vs. male)	0.50** [0.31; 0.80]
Full‐time (vs. part‐time)	0.57** [0.38; 0.86]
*Number of observations*	710
Likelihood ratio test	χ^2^(11) = 185.94, *p* < 0.001
Wald test	χ^2^(11) = 140.36, *p* < 0.001
Estimation method	Estimation method

*Note*: *p < 0.05, **p < 0.01, ***p < 0.001.

Abbreviations: CBI: Copenhagen Burnout Inventory; CI: confidence interval; OR: odds ratio.

In the next step, we undertook granular analyses for physician career stages, respectively (Table 3). We observed that the association between burnout scores and ITLCH was most pronounced in the earlier stages of the medical career. Specifically, burnout scores were significantly associated with increased odds of ITLCH among residents, specialists, and consultants (OR = 1.03, *p* ≤ 0.005). Among consultants with executive responsibilities and chief physicians, the association between burnout scores and ITLCH was weaker and not statistically significant. For consultants without executive responsibilities, age effects occurred (lower odds at 51–60 years, OR = 0.40; significantly higher odds at 61–70 years, OR = 8.51). Female sex and full‐time employment were associated with lower ITLCH (ORs 0.23, 0.47), respectively.

**TABLE 3:** Multivariable logistic regression models for the association between burnout and ITLCH, stratified by the career stage.

	Resident	Specialist	Consultant	Consultant w. exec. resp.	Chief physician
Predictor	OR (95% CI)	OR (95% CI)	OR (95% CI)	OR (95% CI)	OR (95% CI)
Intercept	1.19 [0.30; 5.03]	0.57 [0.01; 112.75]	0.72 [0.23; 2.17]	0.37 [0.02; 5.51]	28.57 [0.43; 12259.34]
Personal burnout score (CBI 3 item)	1.03*** [1.02; 1.05]	1.03** [1.01; 1.06]	1.03*** [1.02; 1.05]	1.02 [0.99; 1.05]	1.01 [0.99; 1.04]
Age 31–40 (vs. ≤30)	0.82 [0.42; 1.59]	0.42 [0.00; 8.90]	—	—	—
Age 41–50 (vs. ≤30 or 31–40)	0.85 [0.15; 5.67]	0.42 [0.00; 9.77]	0.85 [0.43; 1.68]	1.02 [0.12; 11.97]	0.05 [0.00; 1.16]
Age 51–60 (vs. ≤30 or 31–40)	0.67 [0.07; 8.59]	0.12 [0.00; 3.16]	0.41* [0.18; 0.88]	0.28 [0.03; 3.60]	0.09 [0.00; 1.91]
Age 61–70 (vs. ≤30 or 31–40)	—	0.23 [0.00; 13.13]	8.51** [2.04; 50.71]	6.13 [0.54; 97.02]	0.79 [0.01; 16.70]
Female (vs. male)	0.54 [0.18; 1.42]	1.76 [0.41; 7.82]	0.23*** [0.10; 0.51]	0.79 [0.23; 2.74]	1.48 [0.47; 4.80]
Full‐time (vs. part‐time)	0.83 [0.39; 1.70]	0.62 [0.20; 1.79]	0.47* [0.25; 0.87]	0.60 [0.15; 2.50]	0.05* [0.00; 0.95]
Number of observations	231	77	226	74	102
Likelihood ratio test	χ^2^(6) = 15.87, *p* = 0.015	χ^2^(7) = 14.98, *p* = 0.036	χ^2^(6) = 44.84, *p* < 0.001	χ^2^(6) = 15.47, *p* = 0.017	χ^2^(6) = 29.27, *p* < 0.001
Wald test	χ^2^(6) = 55.73, *p* < 0.001	χ^2^(7) = 13.36, *p* = 0.064	χ^2^(6) = 38.29, *p* < 0.001	χ^2^(6) = 17.48, *p* = 0.008	χ^2^(6) = 27.39, *p* < 0.001
Estimation method	Firth's penalized likelihood (profile likelihood)	Firth's penalized likelihood (profile likelihood)	Firth's penalized likelihood (profile likelihood)	Firth's penalized likelihood (profile likelihood)	Firth's penalized likelihood (profile likelihood)

*Note*: *p < 0.05, **p < 0.01, ***p < 0.001.

Abbreviations: CBI: Copenhagen Burnout Inventory; CI: confidence interval; ITLCH: intention to leave current hospital; OR: odds ratio.

Further analyses suggested that (i) the burnout‐ITLCH association persists when the oldest stratum is excluded, (ii) the association is amplified among full‐time consultants, and (iii) apparent age effects diminish when workload patterns are accounted for—together implying that burnout is an age‐robust correlate of ITLCH, while age and working hours mark different late‐career trajectories (stability vs. transition).

## 4 DISCUSSION

In this nationwide cross‐sectional study, half of the OBGYN physicians working in German hospitals reported moderate to high burnout scores and ITLCH. Burnout was associated with ITLCH, especially among junior and mid‐career physicians.

The burnout prevalence of around 50% in this cohort aligns with existing literature reporting rates between 40% and 75%.^9^ Variability in burnout estimates is well known and attributable to differences in study quality, definitions, and assessment tools.^22^ In benchmarking against the COPSOQ database from the Freiburg Research Center for Occupational Sciences (FFAW) from 2020 to 2024, which includes responses from more than 1500 physicians and 250 000 individuals from the general workforce, mean burnout levels in our cohort (50.6; SD = 21.1) closely matched scores of physicians in Germany (51.4; SD: 21.1) and the general working population (49.9; SD: 21.3).

Consistent with previous research,^5^, ^12^ burnout showed a robust association with ITLCH. Although an ITL is not a perfect proxy for subsequent turnover,^2^ it captures dissatisfaction and early disengagement, which represent important organizational warning signals. Given the substantial financial and operational consequences of physician departures,^3^ burnout represents a critical and modifiable target for retention efforts.

Higher burnout levels among physicians earlier in their careers are consistent with previous findings^14^, ^23^ and have been linked to gender composition,^24^ work–life conflict,^25^ work‐related stressors,^26^ and caregiving responsibilities.^25^ In some countries, educational debt is another burden,^7^ though less relevant in Germany. Challenges evolve over the course of a physician's career,^16^, ^27^ and some studies report higher burnout with age and length of service.^28^ Whether the increased susceptibility to burnout in younger physicians reflects cohort‐specific stressors or general age‐related vulnerability remains unclear.^17^ Studies across different cultural contexts regularly report a decline in mental wellbeing and/or an increase in burnout during residency training, indicating increased vulnerabilities and specific stressors in early‐career physicians,^29^, ^30^, ^31^ likely reflecting an adverse combination of high workload, limited job control, as well as higher expectations and insufficient coping strategies.

Although changing hospitals, particularly after the completion of specialist training, is common and even encouraged by institutions and pursued by individuals for career development, our results indicate that higher burnout scores are associated with increased odds for ITLCH among junior and mid‐career physicians. While workforce stability is generally considered beneficial, supporting quality of care and reducing recruitment costs,^32^ changing workplaces can serve as an effective strategy for leaving burnout‐prone work environments at the individual physician level. For healthcare organizations, implementing targeted strategies to mitigate burnout during these career stages may represent an effective approach for reducing physician turnover associated with burnout.

Among consultants with executive responsibilities, the relationship between burnout and ITLCH was not significant. Age‐stratified analyses suggest diverging late‐career trajectories: physicians aged 51–60 reported low ITLCH, whereas those aged 61–70 showed higher ITLCH, likely reflecting anticipated retirement transitions. Because our outcome measure combined organizational departure and potential exit from clinical practice (“no longer working in the medical sector”), future studies should disentangle these pathways, as higher burnout has been linked specifically to early retirement intentions.^33^ Post‐hoc, the absence of a burnout–ITLCH association in senior physicians may reflect increased autonomy, greater psychological resilience, stronger organizational embeddedness, or a survivor effect in which physicians less able to cope with chronic stress leave the profession earlier.^27^ Indeed, senior physicians tend to report higher career satisfaction, better work‐life balance,^34^ and lower stress from workplace stressors,^26^ suggesting enhanced overall resilience and favorable mental health in advanced career stages. Nonetheless, whether burnout directly drives retirement decisions in senior physicians remains unclear and warrants further investigation.^33^

These findings underscore the need for age‐ and career‐sensitive strategies to support well‐being and retention. While many interventions focus on individual coping strategies, evidence favors structural and organizational approaches.^6^ Excessive workload, compromised professional integrity, and impaired teamwork have been closely tied to burnout and turnover intentions.^35^ Conversely, organizations characterized by effective teamwork and leadership and a culture of personal appreciation are associated with lower burnout and reduced ITL.^36^ These organizational dimensions are modifiable and offer actionable targets for OBGYN departments seeking to stabilize their workforce. In high‐stakes clinical settings, fostering psychological safety may additionally be a key consideration, as teams where staff feel able to speak up about mistakes or concerns without fear of negative repercussions promote learning, well‐being, and retention.^37^ However, a meta‐analysis found little to no practical significance of interventions aimed at reducing burnout among resident physicians, rendering assumptions that individual factors are amenable to sustainable change in provider burnout.^38^

This study offers several strengths. It provides a detailed assessment of burnout and ITLCH among OBGYN physicians across career stages, using a large, nationwide sample. The modeling approach using Firth's penalized likelihood reduced small‐sample bias, particularly in subgroups with low event frequencies.

Several limitations merit consideration. The cross‐sectional design precludes causal inferences, and longitudinal research is needed as burnout and ITL can evolve over time.^4^ The directionality and persistence of these associations remain uncertain. In addition, recent acute events may have influenced the single‐time‐point measurement and, therefore, may not necessarily reflect a chronic state of burnout. On the other hand, traumatic events as well as post‐traumatic stress symptoms – which are common among OBGYN practitioners^13^ – can contribute to mental health problems that may not be adequately addressed by interventions targeting work or personal life alone.

Second, although the CBI is a validated and widely used instrument, agreement between CBI and other burnout measures (e.g., Maslach Burnout Inventory) may vary, particularly in younger physicians or trainees.^39^ This may introduce measurement variability and limit comparability to other international studies using different tools.^22^ Both burnout and ITLCH were self‐reported, introducing potential common‐method bias. Additionally, unmeasured personal and professional factors such as caregiving responsibilities^25^ and professional fulfillment^12^ may influence both outcomes. Furthermore, the voluntary nature of participation introduces a risk of selection bias, which may limit generalizability. Physicians experiencing higher levels of burnout or dissatisfaction may have been more likely to respond, whereas those who are more satisfied or less stressed may be underrepresented.

Finally, representativeness may be limited. For benchmarking purposes, we compared our sample with official German hospital statistics.^40^ In these statistics, physicians are categorized by hierarchical position (chief physicians, consultants, residents), whereas board certification (specialist status) is recorded separately and not used as a primary classification criterion. This implies that specialists may be classified as either consultants or residents. Furthermore, workforce data are only available for OBGYN departments as a whole (*n* = 691), rather than exclusively for OB departments providing solely obstetric care (*n* = 595).

For 2024, a total of 741 chief physicians (500 male), 3174 consultants (1019 male), and 2276 residents (504 male) are reported. Based on these figures, the estimated overall response rate is 14.1% for OBGYN physicians in Germany. The sample included a higher proportion of physicians in executive roles as well as a greater proportion of female respondents, approximately 10% above the national average.^40^ Given known gender differences in occupational stress, this may have affected estimated burnout levels. Overall, due to the limitations outlined above, a response rate can only be approximated. This restricts the generalizability of our findings and may result in an overestimation of burnout prevalence and a potentially inflated association with the ITLCH.

## 5 CONCLUSION

The association of burnout with intention to leave the current hospital differs along different career stages among OBGYN physicians, with junior and mid‐career physicians showing significantly positive associations. Our findings corroborate the need for targeted interventions to improve retention, particularly among residents and younger OBGYN physicians.

## AUTHOR CONTRIBUTIONS

Conceptualization: Arno Stöcker, Nadine Scholten, Matthias Weigl; Data Curation: Anna Volkert, Malte Bäckmann; Formal Analysis: Arno Stöcker; Funding Acquisition: Nadine Scholten; Investigation: Nadine Scholten, Anna Volkert, Mi‐Ran Okumu, Malte Bäckmann, Matthias Nübling; Methodology: Arno Stöcker, Nadine Scholten; Project Administration: Nadine Scholten, Anna Volkert, Mi‐Ran Okumu; Supervision: Nadine Scholten; Validation: Malte Bäckmann; Visualization: Arno Stöcker; Writing – Original Draft Preparation: Arno Stöcker; Writing – Review & Editing: Nadine Scholten, Matthias Weigl, Anna Volkert, Mi‐Ran Okumu, Malte Bäckmann, Matthias Nübling, Brigitte Strizek.

## FUNDING INFORMATION

The project MAM‐Care is funded by the Federal Ministry of Research, Technology and Space (grant no. 01GY2110). Nadine Scholten received the grant.

## CONFLICT OF INTEREST STATEMENT

All authors declare that they have no conflicts of interest.

## ETHICS STATEMENT

Ethical approval was granted by the Ethics Committee of the Medical Faculty, University of Cologne (22‐1260) on February 9, 2023. Participation in the anonymous survey was considered to imply informed consent upon return of the completed questionnaire or submission of the online survey.

## Data Availability

The data that support the findings of this study are openly available in OSF at https://osf.io/vxc7h/overview?view_only=aa777033768943f889f78916393befb1.
